# QUALITY-OF-LIFE INDICATORS IN CAREGIVERS OF OLDER ADULTS WITH DEMENTIA: CATEGORIZATION USING THE WHO-ICF

**DOI:** 10.1093/geroni/igad104.2553

**Published:** 2023-12-21

**Authors:** Idorenyin Udoh, Elias Mpofu

**Affiliations:** University of North Texas, Denton, Texas, United States; University of North Texas, Denton, Texas, United States

## Abstract

The quality of life of caregivers of persons with dementia greatly influences their health, physical, psychological, and emotional wellbeing. This in turn influences the quality of care they deliver to persons with dementia. This scoping review aimed to map emerging evidence on the quality of life indicators using service and environmental support factors and a subsequent characterization using the WHO-ICF. The WHO-ICF provides a platform for the understanding of this factors and how it influences the overall wellbeing of caregivers. Using a systematic scoping review approach, we identified 14 studies on service and environmental support influencing quality of life of caregivers of persons with dementia. The search included EMBASE, CINAHL, the Cochrane Database of Systematic Reviews, JBI Evidence Synthesis, and Epistemonicos. A narrative synthesis of the data revealed that the quality of life of caregivers is greatly influenced by service and environmental support factors which in turn influences their activities of daily living and that of persons with dementia. The service and environmental services factors include services from government, family, community, healthcare professionals, peer groups, and activities available, Attitudes from individuals especially family, spouse and others , environmental support and support relationships improving quality of life of caregivers of persons with dementia requires an understanding of the prevailing service and environmental support which drive their commitment to quality care for older adults with dementia especially in a pandemic environment. Keywords: Caregiver, dementia, service, environmental support, quality of life.

